# Effects of Self-Empowered Teams on Rates of Adverse Drug Events in Primary Care

**DOI:** 10.1155/2012/374639

**Published:** 2012-02-16

**Authors:** Ranjit Singh, Diana Anderson, Elizabeth McLean-Plunkett, Angela Wisniewski, Renee Kee, Kelvin Gold, Chet Fox, Gurdev Singh

**Affiliations:** ^1^Department of Family Medicine, State University of New York at Buffalo, Buffalo, NY 14203, USA; ^2^UB Patient Safety Research Center, State University of New York at Buffalo, Buffalo, NY 14203, USA; ^3^School of Management, State University of New York at Buffalo, Buffalo, NY 14215, USA

## Abstract

*Background.* Most safety issues in primary care arise from adverse drug events. Team Resource Management intervention was developed to identify systemic safety issues to design and implement interventions to address prioritized issues. *Objectives.* Evaluate impact of intervention on rates of events and preventable events in a vulnerable population. *Design.* Cluster randomized trial. 12 practices randomly assigned to either: (1) Intervention; (2) Intervention with Practice Enhancement Assistants; (3) No intervention. The intervention took 12 months. *Main Outcome Measure.* Rate and severity of events and preventable events measured using a Trigger Tool chart review method for the 12-month periods before and after the start of the intervention. *Results.* In the ‘‘intervention with Assistants” group there was a statistically significant decrease in the overall rate of events and in the rate of moderate/severe events. Analysis of Variance with study arm and time as the factors and moderate/severe events as the outcome showed a significant interaction between arm and time supporting the notion that the ‘‘Intervention with Assistants” practices had a greater reduction in moderate/severe preventable events. *Conclusions.* The intervention had a significant effect on medication safety as estimated using a trigger tool. Further exploration of role of Assistants and trigger tool is warranted.

## 1. Introduction

Medication use is recognized to be a high-risk activity across all settings. A recent Institute of Medicine (IOM) report on this subject acknowledges that the rates and impact of medication errors are huge but are poorly understood [1]. The President of the Institute of Safe Medication Practices (ISMP), Michael Cohen [2], in his testimony to a committee of the US Congress estimated that the dollar cost of adverse drug events was about $200 billion across all settings. In ambulatory settings, medication errors and adverse drug events (ADEs) are one of the most important safety issues. A study based on the National Ambulatory Medical Care Survey (NAMCS) found that office-based physicians prescribed at least 1 inappropriate medication to nearly 8% of the elderly who received prescriptions [3]. Another study of ambulatory elderly patients with polymorbidity and associated polypharmacy documented that 35% reported experiencing at least one ADE within the previous year [4]. Gurwitz and colleagues have estimated (by extrapolation) that Medicare enrollees alone suffer approximately 500,000 preventable ADEs per year [5].

A 2003 report of a multidisciplinary group (composed of the AHRQ-supported Medical Group Management Association Center for Research, the Centers for Medicare and Medicaid Services, and the Partnership for Patient Safety) draws attention to the fact that while safety risks are widespread in ambulatory settings, there has been insufficient attention paid to them [6]. Some estimates suggest that ambulatory settings are at least equally important as inpatient settings, with up to 200,000 avoidable deaths annually in the United States of America (USA) alone [7, 8].

Lack of awareness of the type, the incidence, and consequences of errors in any setting is one of the most important barriers to reducing these errors and improving quality of care. The most commonly used method for estimating vulnerabilities in healthcare is to *retrospectively *collect and count errors through voluntary reporting systems (often referred to as “incident reports”). These are fraught with difficulty due to underreporting (according to IOM's 1999 report, only 5% of known errors are typically reported—and then there are unknown errors) and abuse (e.g., reports filed and counterfiled as a means of retaliation against colleagues [9]). Error reporting often does not promote understanding of the organizational structure and processes of care. Instead it tends to be associated with blame and shame, and frequently results in antagonism between team members undermining mutual respect, trust, and cooperation. The Patient Safety and Quality Improvement Act (2005) was introduced in large part to stimulate increased error reporting through the creation of Patient Safety Organizations (PSOs). Bates and colleagues have described difficulties involved in defining and quantifying errors; they report that even direct observational studies, which are highly labor intensive, often miss errors [10]. An alternative approach that is *prospective*, rather than retrospective, and encourages involvement of all teammembers for identifying and prioritizing safety and quality problems invokes Failure Modes and Effects Analysis (FMEA). This has been widely used in other high-risk industries and has been advocated by the IOM^9^ as a means of analyzing a system to identify its weaknesses (“Failure Modes”), possible consequences of failure (“Effects”), and to prioritize areas for improvement. We have adapted and tailored this methodology to allow for the levels of resources and expertise available in ambulatory settings, and developed an instrument that has been shown to be effective in a variety of these settings. The details of the rationale and processes behind this instrument termed ‘‘Safety Enhancement and Monitoring Instrument that is Patient Centered” (SEMI-P) are described elsewhere [11–15].

It is important to recognize, as Crabtree et al. [16] have done, that a vital step toward creating the Medical Home is to close the physician staff divide so as to maintain communication and coordination. They draw attention to the fact that currently practice meetings are “universally unpopular” despite their indispensability. Our TRM methodology, invoking the paradigm of complex adaptive systems, is designed to aid formation of central “attractors” in the form of self-empowered effective learning teams with a common vision to help their complex microsystems to adapt and thrive [17–21]; thriving systems are endowed with simple rules, shared vision, and opportunities for team members to innovate.

An outcome-oriented team has to be enabled to (a) own and identify vulnerabilities in their settings, (b) design and implement interventions tailored for its settings, (c) monitor the efficacy of these interventions, and (d) continue the never ending journey in pursuit of safe care, that is, continuing quality improvement. Interestingly, a recent study by Quinn et al. [22] found that physicians from practices that were involved in evaluation of quality improvement activities had significantly less isolation, stress, and dissatisfaction.

**Objective**

The primary objective of this study was to evaluate the impact of the *prospective* team resource management methodology (TRM), based on the SEMI-P instrument, (with and without the use of a practice enhancement associate (PEA)) on

- the number of preventable ADEs in a vulnerable population aged 65 and above;
- the severity of these preventable ADEs.

## 2. Methods

### 2.1. Study Design

This was a cluster randomized trial in which 12 practices in the Upstate New York Practice-based Research Network were each randomized to one of 3 states (4 practices each): (1) team resource management intervention; (2) team resource management intervention with PEA; (3) no intervention (comparison group). Randomization was performed by an independent party, with concealed allocation.

### 2.2. Intervention

**Figure 1:**
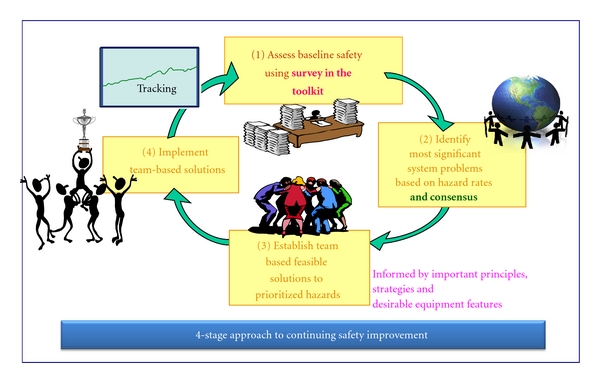
Cyclical Approach to Monitoring and Enhancing Patient Safety.

This team-based approach has four stages. These can be repeated to make a cycle, as shown in Figure 1, for continuous quality and safety improvement.

### 2.3. Stage 1: Safety Enhancement and Monitoring Instrument That Is Patient Centered (SEMI-P)—Duration: 1 Month

**Figure 2:**
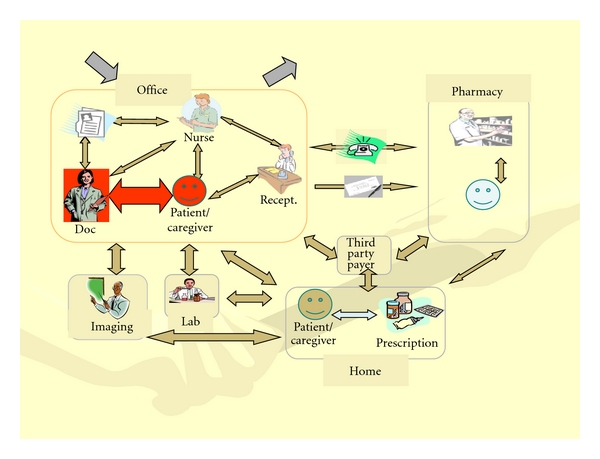
Micromodel of a Medication management in the Ambulatory Setting.

This anonymous survey is an opportunity for *all* staff to freely express opinions about the care processes in their setting. This survey asks about each of the steps in the medication use process. To help orient staff to the overall process, the survey uses a diagram (Figure 2) that shows who and what is involved in the processes and how they work (or are supposed to work) together.

Errors can occur at any point in the process diagram. The survey looks at all the main areas in the process. Each page of the survey is about a different area and consists of a list of errors or causes of error that can occur in that area. The lists are based on review of the literature and consultation with practicing physicians and nursing leaders. The survey asks staff to think about each of the errors in turn and, for each, to indicate their opinion about how often it occurs and, when it does happen, how severe the consequences usually are. The bottom of each page provides an explanation of the various options. The diagram of the testing process is included on each page, with red highlighting to show which part of the process is being asked about.* Everyone *who works in the practice is asked to complete the survey anonymously. If willing, they can mark their job category (provider, nursing, or administrative). The respondents took an average of 20 minutes to complete the survey.

### 2.4. Stage 2: Identification of Most Hazardous Problems in the Testing Process, and Choosing Priorities—Duration: 1 to 2 Months

Scores (called Hazard scores obtained by multiplying frequency of each error with its respective severity of consequence) are calculated for each error in the survey, based on respondents' answers to the frequency and severity questions. The items are then listed in rank order (highest to lowest) and presented to staff in a graphical format in a group meeting for their discussion. This helps the team to form a common vision and consensus regarding which problems need to be addressed first.

### 2.5. Stage 3: Development of Solutions—Duration: 2 to 3 Months

In further group meetings, staff discuss the prioritized problems and work together to design solutions, keeping in mind the resources available and the capabilities of their unique setting.

### 2.6. Stage 4: Implementation and Tracking of the Solutions—Duration: 6 to 8 Months

Teams are formed to implement the solutions, with clear allocation of responsibilities and an agreed time schedule. Solutions are revised based on experience.

All staff at the intervention sites were invited and encouraged to participate. The total intervention period was 12 months.

### 2.7. Role of the PEA

One PEA was assigned to work with all 4 practices in this group. She had completed a Master's degree in nutrition but with no prior experience working with practices. She participated in all study-related team meetings at each practice and made herself available to each practice for up to half a day a week throughout the study period to support safety and quality improvement activities (not limited to study-related activities). The main contribution of the PEA was to follow through on plans developed by the practice team, that is to support Stages 3 and 4 in the TRM cycle outlined above. Examples of PEA activities include developing patient education materials, preparing draft protocols, and collecting resources for patient prescription assistance.

### 2.8. Outcome Measure

The rate and severity of ADEs and preventable ADEs were measured by chart review for the two 12-month periods before and after the start of the intervention. Eligible patients were those aged 65 years and above, who had at least one visit for cardiovascular disease (ICD-9-CM codes 390–459) during the measurement period and at least one visit for any diagnosis in the prior year. If there were 200 or fewer eligible charts at a site, all were screened, otherwise a random sample of 200 was taken.

The chart review was performed using a Trigger Tool method that involves 2 steps, namely a screening step and a review step [5, 23–32]. The screening step involves identifying the presence of certain chart findings, known as triggers, that are known to be possible evidence of an adverse event. In this study, the triggers of interest are those that might represent an adverse drug event (ADE). Examples include an elevated INR (often associated with adverse effects of warfarin) and abrupt discontinuation of a medication (that sometimes occurs because of an ADE). In our study research assistants performed the screening step, using a previously published ADE trigger tool [23] that we adapted from the work of others [5, 25, 26]. In the second step, known as the review step, a physician and pharmacist reviewed the identified triggers to determine for each trigger: (1) whether an ADE took place and if so, (2) whether the event was preventable, (3) the stage of the medication use process where the ADE originated (prescribing, dispensing, administration, and monitoring), and (4) the effect on the patient (none/minimal, mild, moderate, or severe). Examples of preventable errors include missed allergy, wrong dosage, errors of dispensing, administration errors, and failure to order or complete laboratory monitoring.

 The physician and pharmacist worked together, reviewing, discussing, and recording their consensus opinion of each trigger. When they were unable to reach consensus a final determination was made by a third reviewer. If they identified any potential or actual harm that had not been previously recognized and addressed, they notified the primary care physician or site medical director. Triggers that were deemed to represent the same ADE were excluded to avoid “double counting” an ADE.

 The study protocol was approved by the Social and Behavioral Sciences Institutional Review Board of the State University of New York at Buffalo.

## 3. Results

Part way into the study, one of the Comparison group practices withdrew from the study, citing concerns over the administrative burden of the chart reviews (the practice was undergoing administrative changes).  Table 1 shows the characteristics of the 11 sites that completed the study.

At baseline, 1970 charts (mean 179 per site) were screened. Of these, 1066 (54.1%) had triggers, yielding a total of 2898 triggers. This far exceeded our expectation and therefore posed a practical problem due to the effort (and therefore cost) associated with reviewing this number of triggers. Therefore, we elected to reduce the sample by randomly eliminating a proportion of patients at each site, sufficient to reduce the number to approximately 100 patients per site. This yielded a total of 1125 patients, of whom 598 patients had one or more triggers, all of which underwent review. A similar process was used to make the endpoint data manageable, yielding 1050 patients, of whom 564 had triggers that were reviewed.

Table 2 summarizes the rates of preventable ADEs (normalized per 100 patient-years) for each arm of the study. For each arm, we compared the pADE rate (per 100 patient-years) at the two time points (After versus Before) by means of a paired *t* test with sites as the unit of analysis. As shown in Table 2, in the “Intervention with PEA” group there was a statistically significant decrease in the overall rate of pADEs after the intervention compared to before (7.4 per 100 patient-years versus 12.6, *P* = 0.018) and in the rate of moderate or severe (combined) pADEs(1.6 versus 6.4, *P* = 0.035). In the Comparison arm and the unaided Intervention arms, there were no statistically significant differences over time.

Analysis of Variance (ANOVA) with study arm and time as the factors and total pADEs as the outcome variable showed no significant interaction between arm and time. For the outcome of moderate or severe pADEs, the interaction term was significant (*P* = 0.023) supporting the notion that the “Intervention with PEA” practices had a greater reduction in moderate/severe pADEs over time than the comparison group.

## 4. Discussion

The TRM approach, when aided by a PEA, demonstrated a significant reduction in pADEs, especially those with the highest severity. The reduction in pADEs represents a significant improvement in patient safety through the collective efforts of practice staff guided by a structured TRM process.

It appears that the additional resource offered in the form of a PEA was important, as the non-PEA practices did not achieve the same statistically significant improvement that the PEA practices did. While the “mechanism of action” of the PEA is not clearly understood, it is reasonable to surmise that the extra human resource represented by the PEA enabled practices to more effectively implement their planned interventions. Practices may generally have difficulty following through on plans because of existing high workloads. The addition of a PEA may have helped to minimize the incremental burden on teams that are already overburdened.

While we did not formally evaluate this, it is the authors' observation that the humanistic self-empowerment approach used in this study helped to energize front-line workers to maintain and continually improve quality. Staff commented that seeing other people's perspectives (as reflected in SEMI-P results) helped to improve mutual understanding, leading to consensus. Further, we believe that by closing the physician staff divide, and encouraging closer communication and coordination between team members in addressing practice issues, the TRM approach can achieve progress toward the creation of the Medical Home [16].

The study was a pilot study and has various limitations. Firstly, the outcome measure is based on a trigger tool methodology that has limited sensitivity. In fact, the sensitivity of the trigger tool is unknown because there is no gold standard against which to compare it. This means that the rates of pADEs reported should not be seen as estimates of true rates. Instead, they represent only a subset of pADEs, that is, those that are identifiable by the trigger tool. However there is no reason to expect that the sensitivity of the trigger tool would vary between study groups or over time so the validity of the study findings is not jeopardized. Another significant weakness is the small size of the study. Having only 3-4 practices in each arm limited the power. A larger study including a greater number of practices is warranted. In addition, further exploration of the role of the PEA is required to establish the most important “active ingredients” so that these can be deployed in an efficient way.

## Figures and Tables

**Table 1:** Characteristics of the study sites.

Site Characteristic	Arm 1			Arm 2				Arm 3			
	Site 1A	Site 1B	Site 1C	Site 2A	Site 2B	Site 2C	Site 2D	Site 3A	Site 3B	Site 3C	Site 3D
Ownership	Hospital (satellite)	Hospital (satellite)	Private	Hospital (satellite)	Private	Private	Hospital (onsite)	Private	Hospital (onsite)	Hospital (onsite)	Private
Geographic location	Urban	Urban	Urban	Urban	Urban	Rural	Urban	Urban	Urban	Urban	Urban
Residency practice site? (Y/N)	Y	Y	N	Y	N	N	N	N	Y	N	Y
Approximate visits per year	13,780	9,300	37,800	25,000	23,000	5,000	5,000	60,000	18,000	4,500	13,000
Total Staff	45	36	60	40	20	3	20	45	82	12	30

**Table 2:** Preventable adverse drug event rates (pADEs).

	Comparison arm		Intervention arm (unaided)		Intervention arm (with PEA)	
Severity of pADE	Before (*n* = 253)	After (*n* = 294)	Before (*n* = 419)	After (*n* = 390)	Before (*n* = 453)	After (*n* = 366)
All Severities	27 (10.7)	25 (8.5)	38 (9.1)	18 (4.6)	56 (12.4)	27 (7.4)*
None/Minimal or Mild	18 (7.1)	12 (4.1)	27 (6.5)	15 (3.8)	27 (5.8)	21 (5.8)
Moderate or Severe	9 (3.6)	13 (4.4)	11 (2.6)	3 (0.8)	29 (6.4)	6 (1.6)**

*P = 0.018 by paired *t*-test (after versus before) at the site level.

**P = 0.035 by paired *t*-test (after versus before) at the site level.

Values presented as number of pADEs (rate per 100 patient-yrs).
